# Bicarbonate and Carbohydrate Co‐Ingestion During Exercise in a Novel Gel Format Improves Time‐Trial Performance After 3 h of Moderate‐Intensity Exercise in Trained Cyclists

**DOI:** 10.1111/sms.70373

**Published:** 2026-09-25

**Authors:** Bernardo R. Norte, Alex Hickman, Antonio Muñoz Megias, Erick Mosquera‐Lopez, Stéphane Bermon, Frédéric Garrandes, Paolo E. Adami, Kelsie O. Johnson, Elizabeth Mahon, Sam O. Shepherd, Juliette A. Strauss, Julien B. Louis

**Affiliations:** ^1^ Research Institute for Sport and Exercise Sciences (RISES) Liverpool John Moores University Liverpool UK; ^2^ Health and Science Department World Athletics Monaco Monaco; ^3^ Precision Fuel & Hydration, Ltd Christchurch UK

**Keywords:** acid–base balance, durability, endurance, ergogenic aid, fatigue resistance, nutrition, physiological resilience, power‐duration relationship

## Abstract

Sodium bicarbonate (SB) ingestion may improve high‐intensity exercise performance in efforts that range from 1 to 60 min; however, to date, no studies have investigated the effects of SB supplementation during exercise, nor its potential to attenuate the fatigue‐induced decline in parameters of the power‐duration curve during prolonged exercise (i.e., durability). This study examined the effects of bicarbonate (BI) and carbohydrate (CHO) co‐ingestion via a novel gel format during exercise on subsequent sprint and time‐trial (TT) performance in cyclists. Eleven trained cyclists took part in this randomized, crossover study. Following one visit to establish a non‐fatigued power profile (3 × 6‐s sprints, 2‐min TT and 12‐min TT cycling performance), participants completed two experimental trials consisting of 3 h cycling at 95% gas exchange threshold and ingested CHO at a rate of 80 g·h^−1^ with (CHO + BI; 18 g of SB and 2 g of potassium bicarbonate) or without BI co‐ingestion (CHO). Participants repeated the same power profiling protocol after the 3 h submaximal exercise. Performance in the 12‐min TT was improved in the CHO + BI condition compared with CHO (+12 W; *p* = 0.01, *g* = 1.07). Mean power output in the 6‐s sprints (+12 W; *p* = 1.00, *g* = 0.18) and 2‐min TT (+6 W; *p* = 0.888, *g* = 0.32) were not significantly different between CHO + BI and CHO. There were no differences in gastrointestinal symptoms between conditions. These data suggest that BI and CHO co‐ingestion during prolonged exercise via a novel energy gel format improves subsequent 12‐min TT performance in a fatigued state with minimal gastrointestinal discomfort.

## 1 Introduction

Performance in road cycling is often modeled using maximal oxygen uptake (V̇O_2max_), lactate thresholds, and exercise economy assessed under well‐rested laboratory conditions [1]. However, these variables may deteriorate during prolonged exercise [2, 3, 4], lowering the capacity to sustain high power outputs after increasing levels of accumulated fatigue. The ability to withstand the functional decline in physiological profiling characteristics over time has been coined durability or physiological resilience [5, 6]. While these terms are used interchangeably in the literature, durability more precisely refers to changes in external power output or speed at anchor points in the power‐duration curve (e.g., lactate threshold, critical power) or mean maximal power (MMPs) profiles, whereas physiological resilience describes the robustness of physiological determinants of performance (e.g., V̇O_2max_, exercise economy, gross efficiency) and changes in underlying physiology in the face of accumulating work done [7]. Given that road cycling races are frequently decided by high‐intensity efforts performed after several hours of fatiguing exercise [8], durability may be a stronger predictor of race success than physiological parameters obtained in a non‐fatigued state. Despite this, research exploring the role of nutritional strategies in preserving durability is still in its infancy. Carbohydrate ingestion during exercise represents the most studied intervention shown to improve durability [9, 10, 11], leaving considerable scope for investigating the potential of ergogenic supplements.

Sodium bicarbonate (SB) is one of the most extensively researched ergogenic aids in sport and has been shown to enhance high‐intensity exercise performance [12, 13, 14]. Under conditions of high hydrogen ion (H^+^) production, and associated decreases in intracellular pH, a dose of SB in the range of 0.2–0.4 g·kg^−1^ body mass can increase buffering capacity [15]. This facilitates the efflux of H^+^ and lactate from contracting muscles by increasing blood bicarbonate (HCO_3_^−^) concentrations within extracellular fluid by 4–8 mmol·L^−1^ [16, 17]. The resulting state of metabolic alkalosis (elevated pH and HCO_3_^−^) reinforces acid–base balance, attenuating exercise‐induced metabolic acidosis and delaying the onset of fatigue during high‐intensity exercise [18]. Whilst the ergogenic effects of SB are well‐documented for maximal efforts ranging from 30 s to 60 min [18], it remains unexplored whether these benefits extend to high‐intensity efforts performed under conditions of accumulated fatigue following prolonged exercise.

The most utilized ingestion strategies of SB are via solution [13], gelatin or enteric‐coated capsules [19, 20], and more recently, mini‐tablets mixed in a carbohydrate hydrogel system [14, 21, 22]. Ingestion protocols range from single acute doses administered 20–180 min before exercise, to multiple‐day loading protocols and individualized timing based on time to peak HCO_3_^−^ [18]. Despite the diversity in ingestion protocols examined to date, nearly all existing evidence is derived from pre‐exercise supplementation strategies [18]. Notably, Dalle et al. [23] demonstrated a ~3% improvement in sprint performance at the end of a 3‐h simulated cycling race when SB was ingested before and during the race. Whether the sole ingestion of SB during exercise, without any prior loading phase, could represent an equally effective and more practical supplementation strategy is yet to be established. In practice, however, the practical delivery of SB during exercise poses a considerable challenge, at least in part due to the delivery formats currently available. Firstly, a solution form of SB lacks palatability and, secondly, capsule format would require swallowing multiple capsules mid‐exercise (20–25 large capsules for a 75 kg athlete), which is logistically difficult and incompatible with the physiological demands of high‐intensity cycling races. All in all, current delivery formats present important practical limitations for the seamless integration of SB into athletes' existing fueling practices during exercise.

Recently, a novel bicarbonate product in an energy gel format (Bicarb Gel 40; MNSTRY, Freudental, Germany) has been made commercially available. This energy gel contains 5 g of bicarbonate (BI; 4500 mg of SB and 500 mg of potassium bicarbonate) alongside 40 g of carbohydrates (CHO) in a 1:0.8 glucose‐to‐fructose ratio. The BI is encapsulated, purportedly facilitating release in the small intestine rather than the stomach, mechanism by which it is believed to improve gastrointestinal (GI) tolerance. Unlike solution or capsule formats, energy gels represent a convenient delivery method that athletes can readily consume without disrupting exercise. If proven effective, this novel format may extend the application of bicarbonate supplementation beyond pre‐exercise loading protocols, offering endurance athletes a practical strategy for races where fatigue resistance (i.e., durability) is critical to performance.

With this in mind, the aim of our study was to examine the effects of BI and CHO co‐ingestion during exercise on sprint and time‐trial (TT) performance following 3 h of moderate‐intensity cycling. We hypothesized that BI co‐ingestion would induce sufficient alkalosis at the end of the 3‐h cycle to attenuate the decline in time‐trial performance to a greater extent than CHO ingestion alone.

## 2 Methods

### 2.1 Participants

A priori sample size was calculated according to our primary outcome variable, using a repeated‐measures, within‐factor analysis of time‐trial (TT) performance (G*Power, v3.1.9.6; Kiel University, Germany). The analysis determined that a sample of 10 would be sufficient to detect changes in performance with an effect size of *f* = 0.45 (*pη* ^*2*^ = 0.17), an alpha value of 0.05, a power of 0.80, and a default correlation among repeated measures of 0.5. The effect size estimate was deliberately more conservative compared with the magnitude of effects previously reported for TT performance in SB research (*pη* ^*2*^ = 0.39) [12]. Therefore, 11 trained cyclists (8 males, 3 females) were recruited to take part in this investigation (Table 1). Inclusion criteria were as follows: free of illness and musculoskeletal injury, no known cardiovascular or metabolic diseases, self‐reported cycling training volume of > 5 h·week^−1^, a V̇O_2max_ of > 45 mL·kg^−1^·min^−1^, regular participation in competitive TT events (e.g., local 10‐ or 15‐mile TTs) or habituated to self‐pacing maximal efforts, accustomed to ingesting high amounts of CHO during exercise (≥ 80 g·h^−1^ at least once per week), and no known menstrual irregularities or use of hormonal contraceptives (females). The female participants were premenopausal and naturally menstruating (menstrual cycle lengths ≥ 21 days and ≤ 35 days). Participants were ineligible for the study if they were chronically taking any intracellular buffering agents or ergogenic aids. All participants completed a physical activity readiness questionnaire (PAR‐Q) and provided written informed consent. This study adhered to the standards outlined in the updated revision of the Declaration of Helsinki and received ethical approval from Liverpool John Moores University's Research Ethics Committee (260106LJMUREC268).

**TABLE 1:** Participant characteristics (*n* = 11).

Characteristics	Males (*n* = 8)	Females (*n* = 3)	Total (*n* = 11)
Age (years)	41 ± 10	30 ± 8	37 ± 10
Stature (cm)	178 ± 5	164 ± 9	174 ± 9
Body mass (kg)	79 ± 7	63 ± 6	75 ± 10
Cycling experience (years)	7 ± 2	4 ± 1	6 ± 2
Training volume (h·wk.^−1^)	9 ± 3	8 ± 2	9 ± 3
Training frequency (no. of weekly cycling sessions)	5 ± 1	5 ± 1	5 ± 1
Power at GET (W)	206 ± 24	152 ± 23	191 ± 34
Power at GET (W·kg^−1^)	2.6 ± 0.2	2.4 ± 0.2	2.6 ± 0.2
Power at OBLA (W)	259 ± 40	194 ± 31	241 ± 47
Power at OBLA (W·kg^−1^)	3.3 ± 0.4	3.1 ± 0.3	3.2 ± 0.4
V̇O_2max_ (mL·kg^−1^·min^−1^)	53.6 ± 4.1	51.1 ± 4.9	53.0 ± 4.5
*W*_max_ (W)	403 ± 42	300 ± 38	375 ± 62
*W*_max_ (W·kg^−1^)	5.1 ± 0.4	4.7 ± 0.2	5.0 ± 0.4
Trial intensity (W)	195 ± 23	144 ± 22	181 ± 32
Trial intensity (W·kg^−1^)	2.5 ± 0.2	2.3 ± 0.2	2.4 ± 0.2

*Note:* Data are presented as means ± SD. V̇O_2max_, maximal oxygen uptake; GET, gas exchange threshold; OBLA, onset of blood lactate accumulation (power at 4 mmol·L^−1^). Training volume/frequency, weekly average in the 2 months preceding the study.

### 2.2 Experimental Overview

**FIGURE 1:**
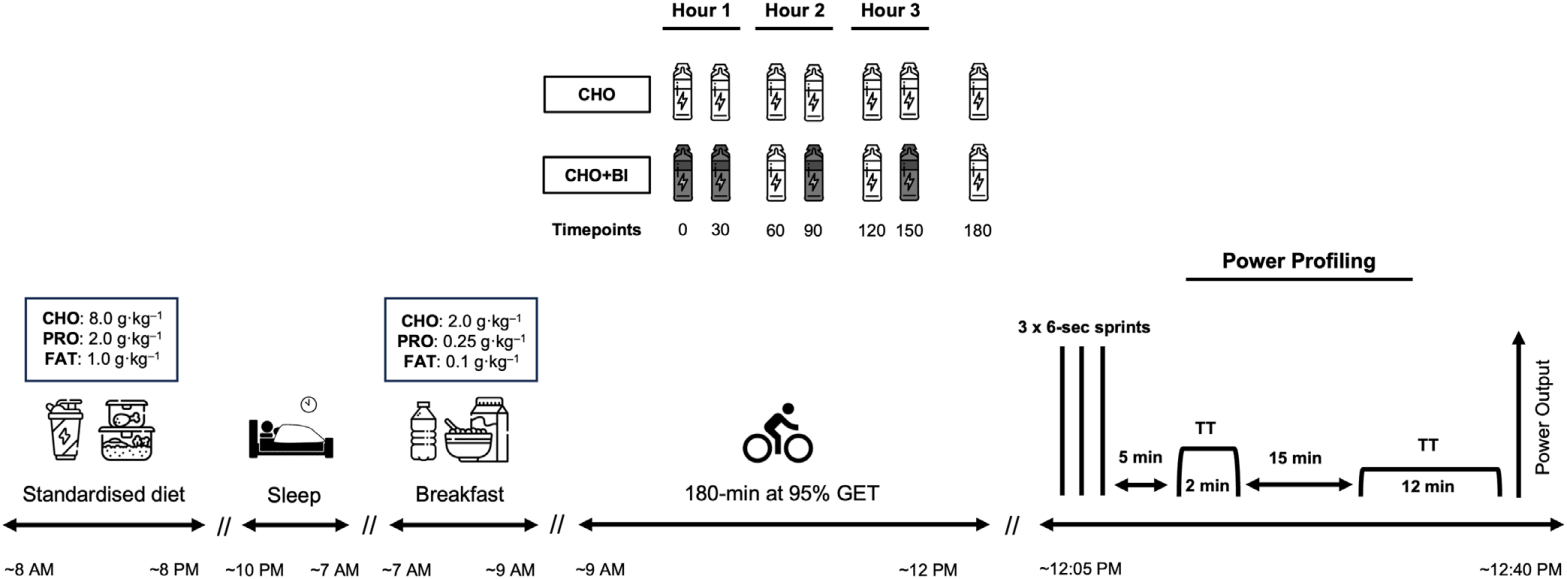
Schematic overview of the experimental trials. Following compliance with a high‐CHO diet, participants consumed a CHO‐rich breakfast before completing a 3 h cycle in the moderate intensity domain, during which they consumed 80 g·h^−1^ of CHO with or without BI co‐ingestion (18 g of SB and 2 g of potassium bicarbonate) in a gel format, followed by a series of power profiling tests in a fatigued state.

This study involved four laboratory visits, adopting a counterbalanced, randomized crossover design, with two characterization trials and two experimental trials, during which participants were provided with 80 g·h^−1^ of CHO (CHO), and 80 g·h^−1^ of CHO with the addition of BI (CHO + BI) in gel format (Figure 1). A full counterbalancing approach was used, generating two possible permutations of the two conditions. These two sequences were each assigned to six participants, resulting in a fully counterbalanced sample of 12 participants. Due to the nature of the delivery format, and the distinct taste and texture profile of the BI in the gel, it was not possible to fully blind participants or the investigator running the experimental trials. Following participant exclusion due to lack of dietary compliance (*n* = 1), the final sample consisted of 11 participants. The first visit was used to establish the baseline physiological characteristics of the participants and perform a familiarization session to the BI gels. The second visit (test day) consisted of a series of power profiling tests (3 × 6‐s all‐out sprints, a 2‐min TT and a 12‐min TT) in a non‐fatigued state. Visits 3–4 were the experimental trials consisting of 180‐min of steady‐state cycling in the moderate intensity domain (at 95% of the estimated GET), followed by the power profiling tests in a fatigued state. Participants were given a standardized dietary plan for the 24 h preceding each experimental trial, consisting of 8.0 g·kg^−1^ CHO, 2.0 g·kg^−1^ protein and 1.0 g·kg^−1^ fat based on individual preferences. The characterization trials were separated by a minimum of 48 h, and experimental conditions were completed 6–8 days apart. The characterization session and experimental trials were performed using the same cycle ergometer (Lode Excalibur Sport, Lode B.V., Groningen, Netherlands), with the power profiling tests in a non‐fatigued and fatigued state being performed on another cycle ergometer (Wattbike AtomX, Nottingham, UK). Expired gas analyses were taken with the same computerized metabolic cart (Vyntus CPX, Vyaire Medical, Höchberg, Germany).

### 2.3 Preliminary Testing

Participants arrived in the laboratory approximately 2.5 h after consuming a CHO‐rich breakfast (1.5 g·kg^−1^ CHO and 0.2 g·kg^−1^ protein), and having refrained from caffeine, vigorous exercise, and alcohol for 24 h. Before beginning the session, body mass and stature were recorded. Participants completed a stepwise incremental protocol on an electrically braked cycle ergometer to identify individual lactate thresholds, followed by a ramp incremental test to determine V̇O_2max_ and the GET, which was subsequently used to set the intensity for the experimental trials. The step test commenced at 90 W (60 W for females) and the intensity increased by 30 W every 4 min thereafter until blood lactate reached a concentration of > 4 mmol·L^−1^, when the test terminated. A capillary blood lactate sample was collected from the fingertip at the end of each stage. Following a 15‐min rest period, participants started the ramp test at 80 W, with the workload increasing by 5 W every 10 s (30 W·min^−1^) until volitional exhaustion or cadence dropped below 70 rpm. V̇O_2max_ was operationally defined as the highest 30‐s V̇O_2_ rolling average recorded during the test, and peak power output (PPO) as the highest 30‐s mean power. GET was determined from the ramp incremental test using the V‐slope method [24]. To account for the lag in the V̇O_2_ response during the incremental test, two‐thirds of the ramp rate (i.e., 2/3 of 30 W = 20 W) were deducted from the power output at GET [25]. The duration of the ramp protocol was 10:31 ± 01:56 min:sec (range: 07:00–12:50 min:sec).

Participants then rested for 20 min before commencing a 1‐h familiarization session to the BI gels where they ingested two gels, each with 40 g of CHO and 5 g of BI, at the beginning (0 min) and halfway (30 min) through the session.

### 2.4 Power Profiling Performed in a Non‐Fatigued State

On the second testing session, participants selected their preferred positions on the cycle ergometer (i.e., saddle and handlebar height and fore‐aft) and this was replicated for both power profiling sessions in a fatigued state. The protocol began with a 15‐min warm‐up at 120 W (90 W for females). Following the warm‐up, participants immediately performed 3 × 6‐s all‐out sprints, interspersed with 1 min of recovery between maximal efforts. Participants then recovered for 5 min at 120 W and commenced the first of two self‐paced TT efforts, the 2‐min TT. After completing the 2‐min TT, participants performed an active recovery of 15‐min at 120 W before starting the second and final self‐paced TT effort of 12‐min. Participants were instructed to average the highest mean power output possible during each TT effort. To ensure optimal pacing, time elapsed and cadence were visible throughout both TTs, but participants were blinded from power output and speed data. During the 12‐min TT, at 4 min intervals, a member of the research team provided standardized reminders instructing participants to pace their effort such that complete exhaustion was achieved by task completion. No verbal encouragement was provided during both TTs.

### 2.5 Pre‐Experimental Controls

Participants completed a 2‐day food diary log using the remote food photography method (RFPM) to estimate habitual dietary intake [26]. Two registered sports nutritionists designed the meal plan to be subsequently used for the experimental trials. In the 24 h prior to each trial, participants refrained from any form of exercise (rest day) and alcohol consumption and consumed the standardized high CHO diet containing 8.0 g·kg^−1^ CHO, 2.0 g·kg^−1^ protein and 1.0 g·kg^−1^ fat. Caffeine consumption was permitted only before 2 pm on the day before each trial and was prohibited thereafter until the completion of the trial the following day. Participants also consumed a standardized CHO‐rich breakfast (2.0 g·kg^−1^ CHO, 0.25 g·kg^−1^ protein and 0.1 g·kg^−1^ fat) approximately 2 h before the commencement of the 180‐min steady‐state cycle. Dietary compliance for each trial was confirmed via RFPM by two members of the research team. Participants were also asked to maintain similar training intensity and volume in the week preceding each trial, and training load (via TrainingPeaks) was confirmed to be comparable between trial weeks. No participant took part in cycling races between trial visits. Finally, participants were asked to sleep for a minimum of 8 h before each trial and to keep bedtime and wake‐up times as consistent as possible between trials, although this was not formally monitored.

### 2.6 Steady‐State Cycle (180 Min) and Power Profiling in a Fatigued State

On the morning of the main experimental trials, participants reported to the laboratory at ~09:00 having consumed the standardized CHO‐rich breakfast described above. Participants completed a 5‐min warm‐up at 100 W and immediately began the 180‐min steady state cycle at 95% GET. Fingertip blood samples were collected at baseline (0 min) and then every 60 min until 180 min using heparinized 90 μL capillary tubes (EPOC Care‐Fill, Siemens Healthineers, Erlangen, Germany) and immediately analyzed for blood pH, HCO_3_^−^, sodium (Na^+^), potassium (K^+^), calcium (Ca^2+^) and chloride (CI^−^) using a blood gas analyzer (EPOC, Siemens Healthineers, Erlangen, Germany). Capillary blood samples (20 μL) were also collected every 30 min and analyzed for blood glucose and lactate concentrations via enzymatic‐amperometric reaction (Biosen C‐Line, EFK Diagnostics, Cardiff, UK). Heart rate (Polar H10, Polar Electro Oy, Kempele, Finland) and rating of perceived exertion (RPE) (Borg 6–20 scale) measurements were taken every 30 min. Expired gases were analyzed for a 4‐min period every 30 min to calculate whole body substrate utilization and total energy expenditure. Participants ingested CHO in energy gel format every 30 min (described below) from 0 to 180 min and GI symptom questionnaires were administered every time upon ingestion of the gel. GI symptoms (nausea, regurgitation, fullness, cramps, flatulence and urge to defecate) were recorded using a 0–10 visual analogue scale (0 = no discomfort and 10 = unbearable discomfort) [27]. Participants were instructed to complete the 11‐point GI scale as follows: 1 to 4 indicated mild GI symptoms (i.e., not substantial enough to interfere with exercise), 5 to 9 indicated severe GI symptoms (i.e., enough to interfere with exercise), and 10 indicated extreme GI symptoms warranting exercise cessation. The cumulative (sum of scores across all symptoms) and peak scores for each individual symptom were calculated for each condition. Perception of sweetness, pleasantness of taste and urge to consume the energy gel were assessed using a modified visual analogue scale every 30 min [28].

Upon completion of the 3‐h cycle, participants dismounted the Lode cycle ergometer and were permitted a toilet break if required. The final energy gel was ingested (180 min), and GI symptom questionnaires were completed before participants rapidly resumed cycling on the Wattbike Atom X (< 3 min transition). Following a 3‐min reacquaintance period at 120 W, participants immediately commenced the fatigued power profiling protocol, beginning with 3 × 6‐s all‐out sprints and thereafter replicating the sequence performed in the non‐fatigued state. During both power profiling sessions in a fatigued state, capillary blood samples were collected to examine changes in blood acid–base balance and electrolyte concentrations at five timepoints: immediately post 3 × 6‐s sprints (PT1), immediately post 2‐min TT (PT2), immediately prior to the 12‐min TT (PT3), immediately post 12‐min TT (PT4), and following 10‐min of passive recovery (PT5).

### 2.7 Carbohydrate and Bicarbonate Ingestion

During exercise, participants consumed CHO at a rate of 80 g·h^−1^, using multiple‐transportable carbohydrates in a 1:0.8 ratio of glucose to fructose. Energy gels contained 40 g of CHO each (Gel 40 Neutral 1:0.8; MNSTRY, Freudental, Germany), with the BI gels containing an additional 5 g of BI (4500 mg of SB, 500 mg of potassium bicarbonate; Bicarb Gel 40 Lemon 1:0.8, MNSTRY, Freudental, Germany). In the CHO only trial, participants consumed one standard CHO gel every 30 min from 0 to 150 min. In the CHO + BI trial, participants ingested a combination of standard and BI gels at predetermined timepoints: BI gels at 0, 30, 90, and 150 min, and standard CHO gels at 60 and 120 min. In both conditions, participants were required to consume 200 mL of water alongside each gel, totalling 1.2 L of water per trial. Additional water intake was permitted *ad libitum* throughout the exercise, and total fluid intake was monitored in both experimental conditions. Body mass was measured before and after the 180‐min cycle to examine differences in fluid loss between trials. Upon completion of the 3‐h cycle, participants ingested a final standard CHO gel (180 min) to maintain adequate carbohydrate availability prior to commencing the fatigued power profiling protocol.

As the BI gels contained a fixed absolute dose of BI, the relative dose was not standardized to body mass (0.27 ± 0.04 g·kg^−1^, range: 0.23–0.35 g·kg^−1^). A fixed absolute dose of 20 g of BI was chosen based on a reference body mass of 70–75 kg, which would correspond to a relative BI ingestion of 0.27–0.29 g·kg^−1^ and consistently shown in the literature to elicit meaningful performance improvements whilst minimizing the risk of GI distress [20]. The distribution of BI gels was strategically determined to optimize the timing of peak HCO_3_^−^ and pH concentrations relative to the fatigued power profiling. Given that time to peak HCO_3_^−^ concentrations after oral SB ingestion is widely varied (~60–180 min), BI gels were front‐loaded to the earlier portion of the ride (0, 30, 90, and 150 min) to ensure a significant level of alkalosis was achieved at the start of the fatigued efforts.

### 2.8 Estimates of Whole Body Substrate Oxidation and Energy Expenditure

Rates of whole body CHO and fat oxidation (g·min^−1^) were measured at 30‐min intervals during the prolonged cycle with the equations of Jeukendrup and Wallis [29] for moderate to high intensity exercise. Total energy expenditure for each trial was estimated assuming an energy yield of 17.57 kJ and 39.33 kJ for 1 g of CHO and fat, respectively.

### 2.9 Statistical Analysis

All data were assessed for normality using a Shapiro–Wilk test and sphericity was assessed using Mauchly's test, with violations corrected via Greenhouse–Geisser. To analyze overall sprint and TT performance, a one‐way repeated measures ANOVA was conducted. A two‐way repeated measures (condition × time) ANOVA was conducted to analyze changes in whole‐body substrate use, RER, total energy expenditure, RPE, heart rate, relative intensity, absolute V̇O_2_, blood lactate and blood glucose during the experimental trials. Where a significant main effect was found, pairwise comparisons were conducted using Bonferroni‐adjusted post hoc tests. Effect sizes concerning ANOVA main effects and interactions were calculated with partial eta squared (*pη* ^*2*^) and interpreted as small (0.01), medium (0.06) or large (0.14) [30]. Due to missing data points, changes in blood acid–base balance (blood HCO_3_^−^ and blood pH) and electrolyte responses (Na^+^, K^+^, Ca^2+^, CI^−^) during the experimental trials were analyzed using a general linear mixed model (LMM), with condition, timepoint and interaction specified as fixed effects and participants as a random intercept. Models were estimated using restricted maximum likelihood (REML), with a compound symmetry (CS) covariance structure across repeated observations. Paired mean differences (MD) are presented with 95% confidence intervals (CI), and effect sizes for ANOVA and LMM pairwise comparisons were expressed as Hedges' *g* and interpreted as trivial (< 0.20), small (0.20–0.49), moderate (0.50–0.79) and large (> 0.80). Paired samples *t*‐tests were conducted to analyze differences in total CHO and fat utilization and cumulative GI symptom scores (sum of all timepoints and GI symptoms combined). Wilcoxon's signed ranks tests were conducted on non‐normally distributed data (i.e., peak GI scores). Non‐normally distributed effect sizes were calculated using *r* (where *r* = *Z*/√ *n*), with 0.10, 0.24, and 0.37 considered as small, medium, and large, respectively [31]. Perceptual ratings of gel sweetness, pleasantness, and urge to consume were analyzed using two‐way repeated‐measures ANOVAs. Although the Shapiro–Wilk test indicated departures from normality at some timepoints, ANOVA was considered appropriate given its robustness to mild violations of normality and the continuous nature of the visual analogue scale data. Finally, the relationship between ΔHCO_3_^−^ (defined as peak HCO_3_^−^ − baseline HCO_3_^−^ at 0 min) and relative BI dose (in g·kg^−1^) in the BI condition was assessed using Pearson's product–moment correlations (*r*). Exploratory analyses of the bivariate relationships between these variables (ΔHCO_3_^−^ and relative BI dose) and Δ12‐min TT (defined as the percentage change in mean power output between CHO + BI and CHO conditions) were also determined. Where presented, percentage change between conditions reflect the means of individual participant differences rather than group‐mean ratios. Data were analyzed using SPSS Statistics (version 30, IBM SPSS Inc., Chicago, IL, USA). All figures were produced using GraphPad Prism 6.07 (GraphPad Software LLC, San Diego, CA, USA). Unless stated otherwise, data in texts, figures, and tables are presented as means ± SD, with *p* < 0.05 denoting statistical significance.

## 3 Results

### 3.1 Power Profiling

**FIGURE 2:**
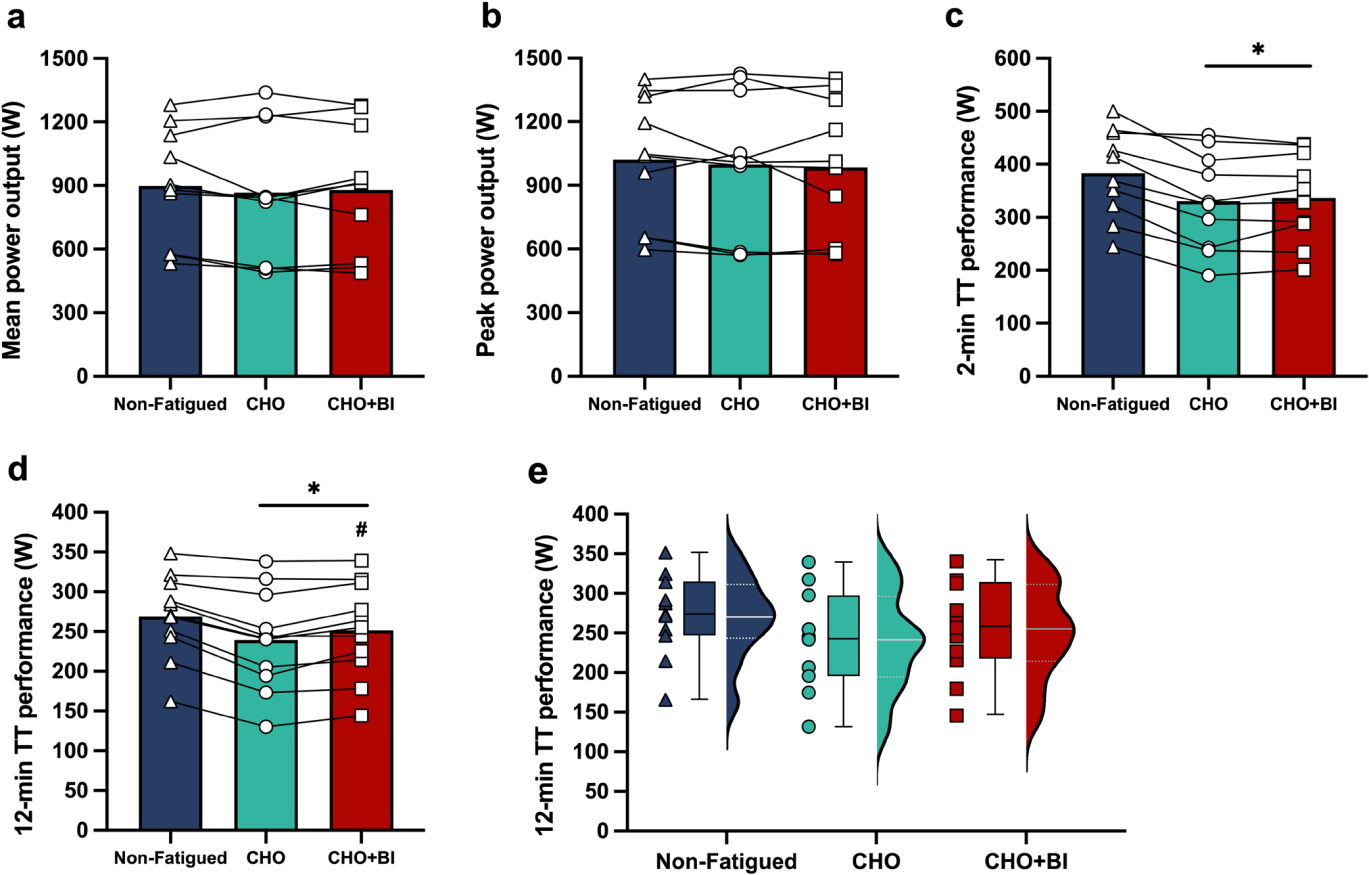
(a) Mean power output (W) in a non‐fatigued and fatigued state following the ingestion of carbohydrates at a rate of 80 g·h^−1^ with (CHO + BI) or without (CHO) the ingestion of BI. (b) Peak power output (W) in a non‐fatigued and fatigued state. (c) 2‐min time‐trial performance (W) in a non‐fatigued and fatigued state. (d) 12‐min time‐trial performance (W) in a non‐fatigued and fatigued state. (e) Raincloud plot depicting individual and group‐level differences in 12‐min time‐trial performance (W) between CHO and CHO + BI conditions. *Significantly different from Non‐Fatigued, *p* < 0.05. ^#^Significantly different from CHO, *p* < 0.05. Bars represent group means, with individual responses shown.

Mean power output in the all‐out sprints was similar between non‐fatigued (Non‐Fatigued: 899 ± 271 W) and fatigued state in both CHO (866 ± 314 W) and CHO + BI (879 ± 304 W) (*p* = 0.371, *pη* ^*2*^ = 0.10; Figure 2a). Likewise, there were no differences in peak power output (*p* = 0.301, *pη* ^*2*^ = 0.12; Figure 2b) in the sprints between Non‐Fatigued (1021 ± 303 W), CHO (998 ± 336 W), and CHO + BI (984 ± 326 W).

Performance in the 2‐min TT was significantly reduced in both experimental conditions (trial effect, *p* < 0.001, *pη* ^*2*^ = 0.80), meaning power output in the CHO (330 ± 91 W, *p* < 0.001, *g* = 1.74) and CHO + BI (337 ± 83 W, *p* < 0.001, *g* = 2.46) conditions were lower than in Non‐Fatigued (383 ± 84 W) (Figure 2c). However, the decline in power was comparable between both conditions (*p* = 0.888). Performance in the 12‐min TT was also significantly affected in both experimental conditions following the 3 h cycle (trial effect, *p* < 0.001, *pη* ^*2*^ = 0.73). There was a significant reduction in power output in the 12‐min TT in both conditions (CHO + BI: 251 ± 53 W; CHO: 239 ± 66 W) compared to Non‐Fatigued (269 ± 55 W, both *p* < 0.05, *g* = 1.15 and *g* = 1.81, respectively), but 12‐min TT performance in a fatigued state was significantly improved following the co‐ingestion of BI and CHO compared to CHO alone (MD: 12 W, 95% CI [3, 22] W, *p* = 0.01, *g* = 1.07) (Figure 2d). A raincloud plot of individual and group‐level differences for the 12‐min TT data can be seen in Figure 2e. There were no trial order (learning) effects for the 2‐min TT or the 12‐min TT (all *p* > 0.05).

Neither ΔHCO_3_^−^ nor relative BI dose were significantly correlated with the magnitude of performance enhancement between conditions for the 12‐min TT. Specifically, there was a nonsignificant positive correlation between ΔHCO_3_^−^ and Δ12‐min TT (*r* = 0.548, *p* = 0.081, 95% CI: −0.077, 0.864), and a nonsignificant positive correlation between relative BI dose and Δ12‐min TT power (*r* = 0.363, *p* = 0.272, 95% CI: −0.302, 0.791).

### 3.2 Blood Acid–Base Balance Analysis

**FIGURE 3:**
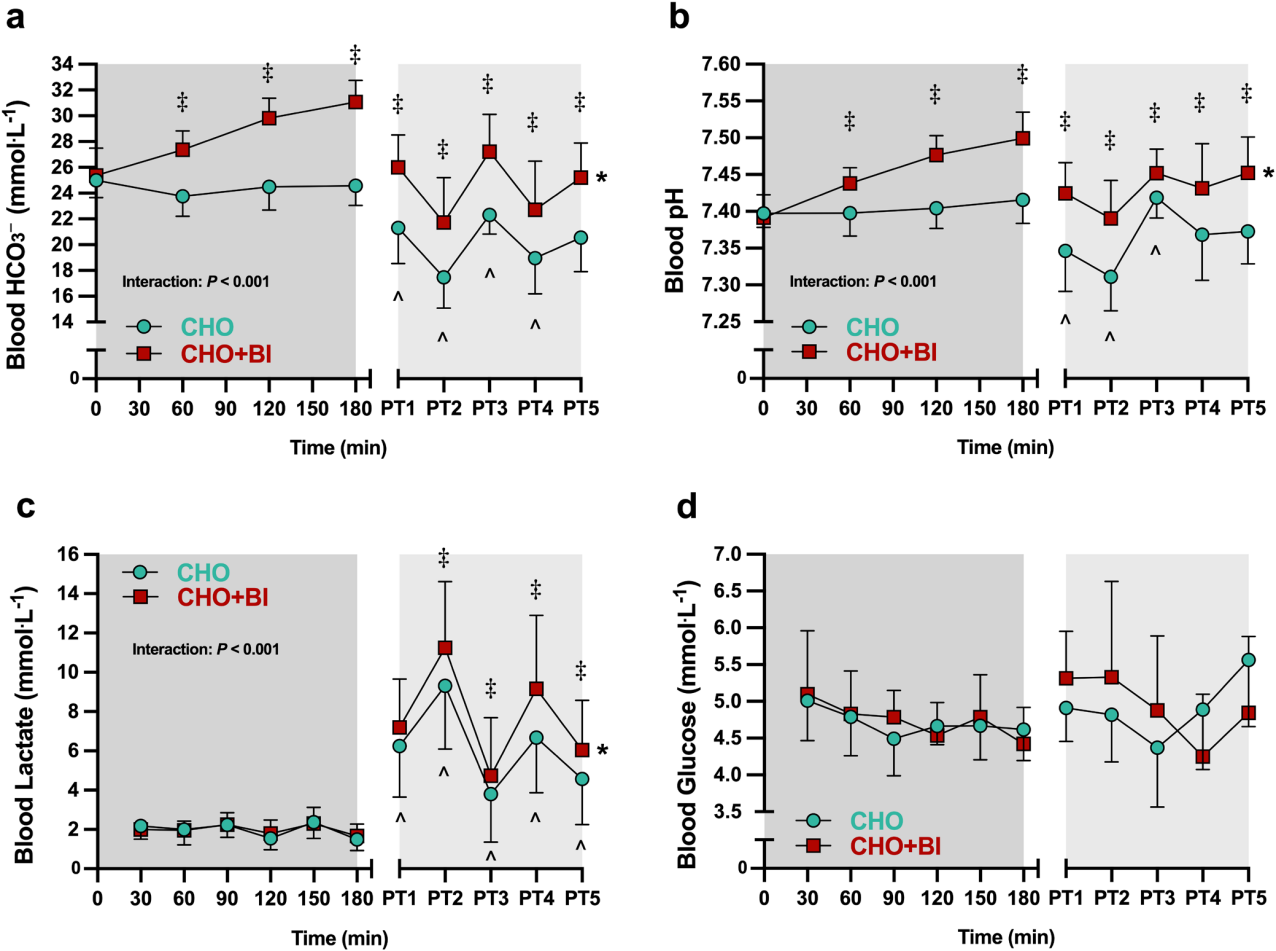
Blood HCO_3_^−^ (a), pH (b), lactate (c) and glucose (d) responses to 180‐min of moderate‐intensity cycling and a power profiling protocol in a fatigued state, with blood sampling taken at 0, 60, 120 and 180 min during the steady‐state cycle, and at post 3 × 6‐s sprints (PT1), post‐2 min TT (PT2), prior to the 12‐min TT (PT3), post‐12 min TT (PT4) and after a 10‐min passive recovery period (PT5). Data are means ± SD for *n* = 11 trained cyclists. *Significantly different from CHO (condition effect), *p* < 0.05. ^‡^Significantly different between conditions at the specified timepoint, *p* < 0.05. ^^^Significantly different from previous timepoint (time effect), *p* < 0.05.

Blood acid–base balance (blood HCO_3_^−^ and blood pH) was similar between conditions at baseline (*p* = 0.743 and *p* = 0.609, respectively). Following the onset of BI supplementation, CHO + BI resulted in significantly higher HCO_3_^−^ concentrations (condition effect, *f* = 201.49, *p* < 0.001; MD: 4.29 mmol·L^−1^, 95% CI: 3.69, 4.89) and pH values (condition effect, *f* = 153.15, *p* < 0.001; MD: 0.06, 95% CI: 0.05, 0.07) compared to CHO, with significant between‐condition differences observed at all timepoints from 60 min through to PT5 (all *p* < 0.005; Figure 3a,b). Both variables also changed significantly over time (HCO_3_^−^: *f* = 37.33, *p* < 0.001; pH: *f* = 19.94, *p* < 0.001), and significant condition × time interactions were observed for both HCO_3_^−^ (*f* = 3.73, *p* < 0.001) and pH (*f* = 4.37, *p* < 0.001). Across both conditions, HCO_3_^−^ concentrations peaked at 180 min (CHO: 24.56 ± 1.53 mmol·L^−1^, CHO + BI: 31.07 ± 1.67 mmol·L^−1^; *p* < 0.001, *g* = 2.64) before declining by approximately 7.4 and 9.3 mmol·L^−1^ at PT2 and 5.6 and 8.3 mmol·L^−1^ at PT4 in CHO and CHO + BI, respectively, coinciding with the post‐TT blood sampling timepoints. HCO_3_^−^ tended to recover during post‐exercise passive rest from PT4 to PT5, although this did not reach significance (*p* = 0.051). In the CHO + BI condition, blood HCO_3_^−^ concentrations were significantly elevated above baseline from 120 min onwards (*p* < 0.001) until the end of the 3 h cycle, but not at 60 min (*p* = 0.706). At the individual level, the majority of participants (9 of 11) reached peak HCO_3_^−^ concentrations at 180 min, with the other two participants peaking at 120 min in the CHO + BI condition. The absolute increase in HCO_3_^−^ from baseline to individual peak concentrations was 5.80 ± 2.17 mmol·L^−1^ (range: 2.80–8.80 mmol·L^−1^) in the CHO + BI condition. A strong positive correlation was observed between ΔHCO_3_^−^ and relative BI dose (*r* = 0.778, *p* = 0.005, 95% CI: 0.335, 0.940), indicating that participants who received a higher relative dose exhibited a greater absolute increase in blood HCO_3_^−^.

Blood lactate responses (Figure 3c) were significantly different between conditions (*f* = 11.85, *p* = 0.007, *pη* ^*2*^ = 0.57) and there was also a significant time‐by‐condition interaction (*f* = 7.97, *p* < 0.001, *pη* ^*2*^ = 0.47), with higher lactate concentrations in the CHO + BI compared with CHO at PT2 (MD: 1.94 mmol·L^−1^, 95% CI [1.00, 2.89], *p* = 0.001, *g* = 1.34), PT3 (MD: 0.93 mmol·L^−1^, 95% CI [0.14, 1.72], *p* = 0.026, *g* = 0.77), PT4 (MD: 2.49 mmol·L^−1^, 95% CI [1.16, 3.81], *p* = 0.002, *g* = 1.23) and PT5 (MD: 1.47 mmol·L^−1^, 95% CI [0.26, 2.68], *p* = 0.023, *g* = 0.79). Although blood lactate remained unchanged across the 180‐min cycle, a main effect of time was also observed (*f* = 34.52, *p* < 0.001, *pη* ^*2*^ = 0.79), with the pairwise comparisons revealing a clear temporal pattern across the power profiling tests. Lactate concentrations rose from PT1 to PT2 (*p* = 0.013), declined during the 15‐min recovery period between PT2 and PT3 (*p* < 0.001), increased again following the 12‐min TT from PT3 to PT4 (*p* = 0.023), and subsequently fell during the 10‐min post‐exercise passive recovery period between PT4 and PT5 (*p* = 0.028).

Blood electrolyte responses (Na^+^, K^+^, Ca^2+^, Cl^−^) concentrations are presented, along with the corresponding results, in Figure S1.

### 3.3 Physiological, Metabolic and Perceptual Responses to Exercise

Blood glucose concentrations (Figure 3d) were similar between conditions (*f* = 0.150, *p* = 0.707, *pη* ^*2*^ = 0.016) and did not significantly change over time (*f* = 2.258, *p* = 0.084, *pη* ^*2*^ = 0.201). There was no condition × time interaction for blood glucose (*f* = 2.748, *p* = 0.061, *pη* ^*2*^ = 0.234).

**FIGURE 4:**
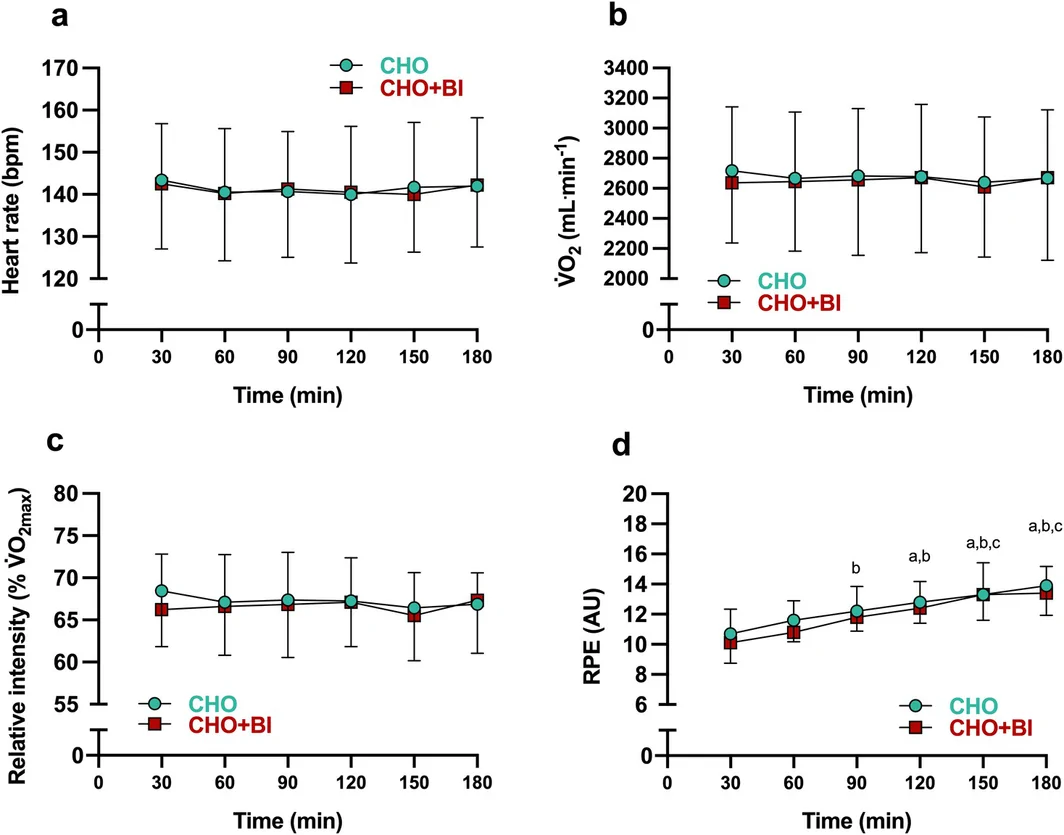
Heart rate (a), absolute oxygen uptake (b), relative intensity (c), and rating of perceived exertion (d) during exercise in the CHO and CHO + BI trials. Data are means ± SD for *n* = 11 trained cyclists. a–c denotes *p* < 0.05 versus ^a^30, ^b^60, and ^c^90 min, respectively.

Heart rate, absolute oxygen uptake and relative intensity did not increase during exercise (time effect, all *p* > 0.05) and did not differ between trials (all *p* > 0.05; Figure 4a–c). Conversely, RPE progressively increased during exercise (time effect, *f* = 20.92, *p* < 0.001, *pη* ^*2*^ = 0.70), with no significant differences between conditions (*f* = 2.08, *p* = 0.184, *pη* ^*2*^ = 0.19; Figure 4d).

### 3.4 Effect of Bicarbonate and Carbohydrate Co‐Ingestion on Whole Body Substrate Metabolism

**FIGURE 5:**
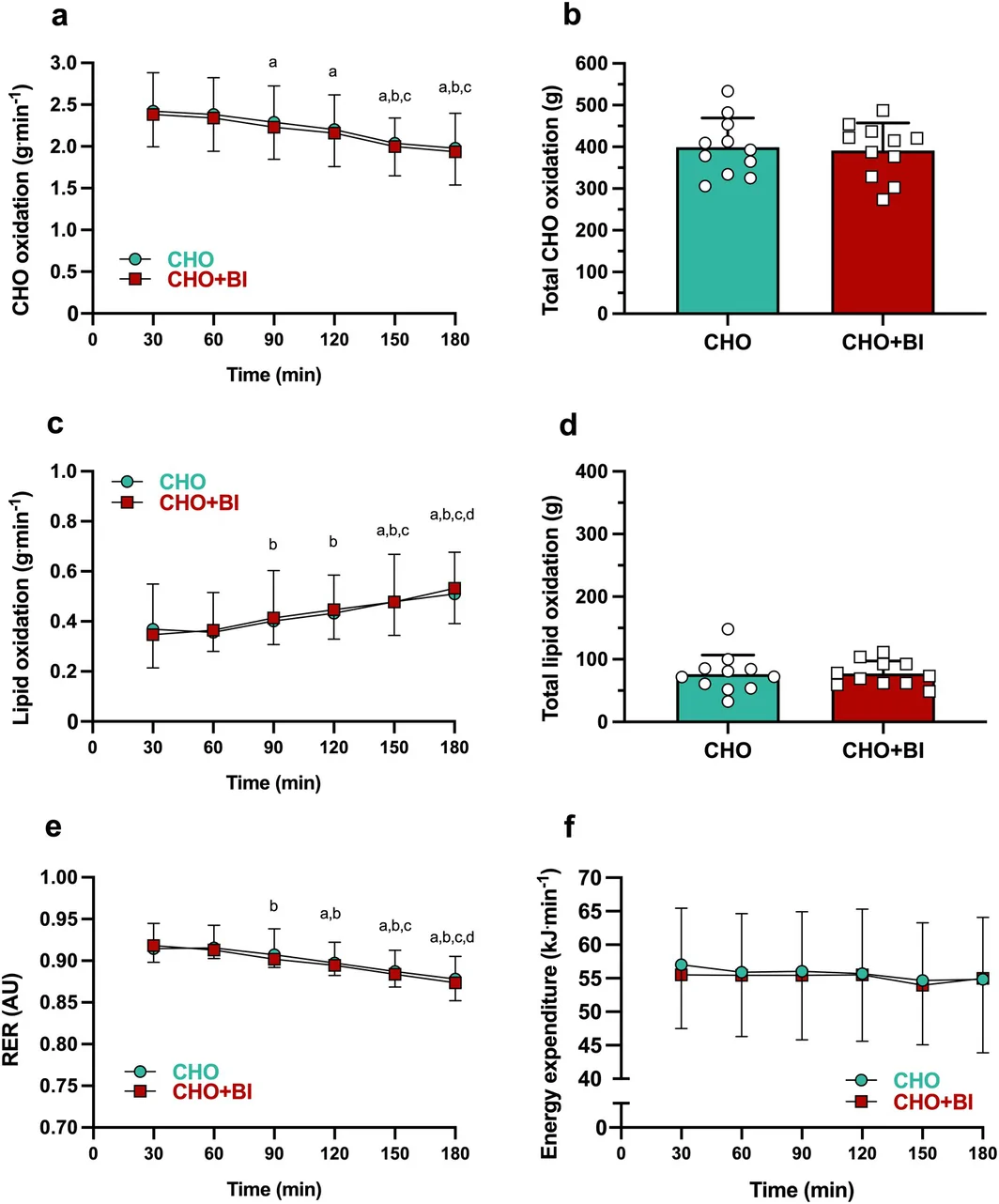
Rates of whole body CHO oxidation during exercise (a), total CHO oxidation (b), rates of whole body fat oxidation during exercise (c), total fat oxidation (d), respiratory exchange ratio (e) and total energy expenditure (f) during exercise in the CHO and CHO + BI trials. Data are means ± SD for *n* = 11 trained cyclists. a–d denotes *p* < 0.05 versus ^a^30, ^b^60, ^c^90, and ^d^120 min, respectively.

Rates of whole body CHO oxidation progressively decreased during exercise (time effect, *f* = 24.06, *p* < 0.001, *pη* ^*2*^ = 0.71) and this was accompanied by a progressive increase in fat oxidation (time effect, *f* = 31.85, *p* < 0.001, *pη* ^*2*^ = 0.76) and a decline in RER (time effect, *f* = 35.48, *p* < 0.001, *pη* ^*2*^ = 0.78). The ingestion of BI during exercise did not influence whole body substrate metabolism during exercise, as evidenced by no significant differences in RER (*f* = 0.18, *p* = 0.677, *pη* ^*2*^ = 0.02), rates of whole body CHO (*f* = 0.94, *p* = 0.354, *pη* ^*2*^ = 0.09) and fat utilization (*f* = 0.04, *p* = 0.850, *pη* ^*2*^ = 0.004), and total CHO (*t* = 0.97, *p* = 0.354, *g* = 0.27) and fat oxidation (*t* = −0.19, *p* = 0.850, *g* = −0.05) between conditions (Figure 5a–e, respectively). Specifically, RER (mean ± SD: 0.90 ± 0.03, 0.90 ± 0.01 AU), rates of CHO oxidation (mean ± SD: 2.22 ± 0.39, 2.17 ± 0.37 g·min^−1^), rates of fat oxidation (mean ± SD: 0.42 ± 0.17, 0.43 ± 0.11 g·min^−1^), total CHO oxidation (mean ± SD: 399 ± 70, 391 ± 66 g) and total fat oxidation (mean ± SD: 79 ± 31, 80 ± 19 g) all displayed similar values between trials. All previous comparisons are reported for CHO and CHO + BI, respectively. Finally, total exercise energy expenditure (Figure 5f) did not differ between trials (condition effect, *f* = 0.64, *p* = 0.441, *pη* ^*2*^ = 0.06) and remained stable over time (time effect, *f* = 1.68, *p* = 0.219, *pη* ^*2*^ = 0.14).

### 3.5 Effect of Bicarbonate and Carbohydrate Co‐Ingestion on Gastrointestinal Discomfort and Gel Palatability

**FIGURE 6:**
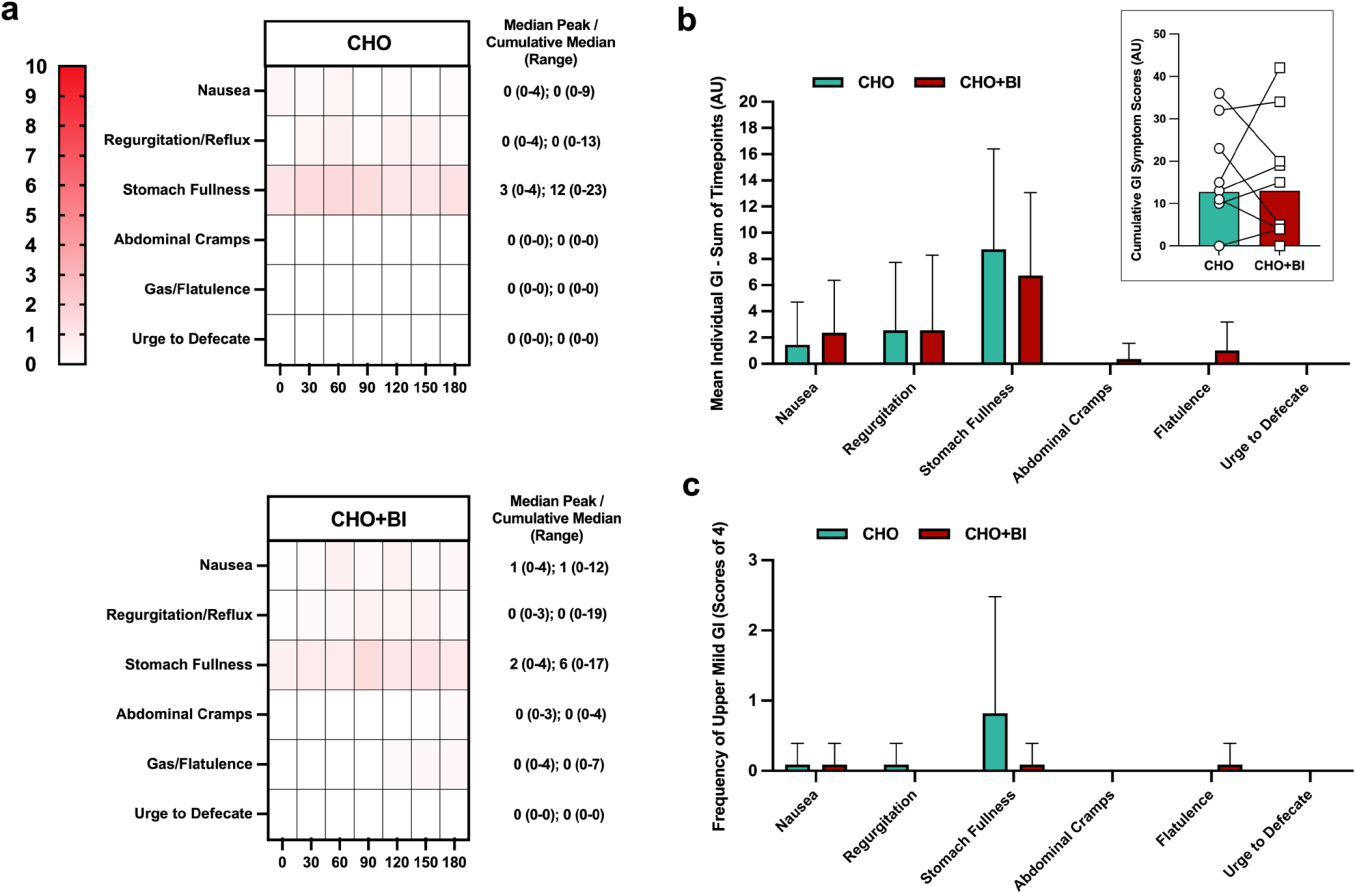
(a) Gastrointestinal symptom prevalence during moderate intensity cycling. (b) Mean GI scores for each individual symptom across each trial (sum of all timepoints), with cumulative GI symptom scores (inset). (c) Frequency of upper mild symptomatology (total number of scores of 4) reported for each individual GI symptom in the CHO and CHO + BI trials.

The incidence of GI symptoms was negligible across both conditions (Figure 6a), with only five participants reporting scores of 4 (upper end of mild GI symptomatology) at any given timepoint for nausea, regurgitation, stomach fullness, and flatulence in either experimental trial. The mean GI scores (sum of all timepoints) for each individual symptom are presented in Figure 6b. Overall, cumulative GI symptom scores (Figure 6b, inset) were similar between conditions (*t* = 0.08, *p* = 0.941, *g* = 0.02). The frequency of scores at the upper end of mild symptomatology (total number of scores of 4 reported at any given timepoint) can be seen in Figure 6c. No participant reported severe or extreme GI symptoms (> 4). Wilcoxon's signed rank tests revealed no significant differences between conditions in peak GI scores for nausea, stomach fullness, abdominal cramps, flatulence and urge to defecate (all *p* > 0.05). Participants experienced no side effects of BI ingestion post‐trials, and no GI disturbances were reported for the remainder of each day.

**FIGURE 7:**
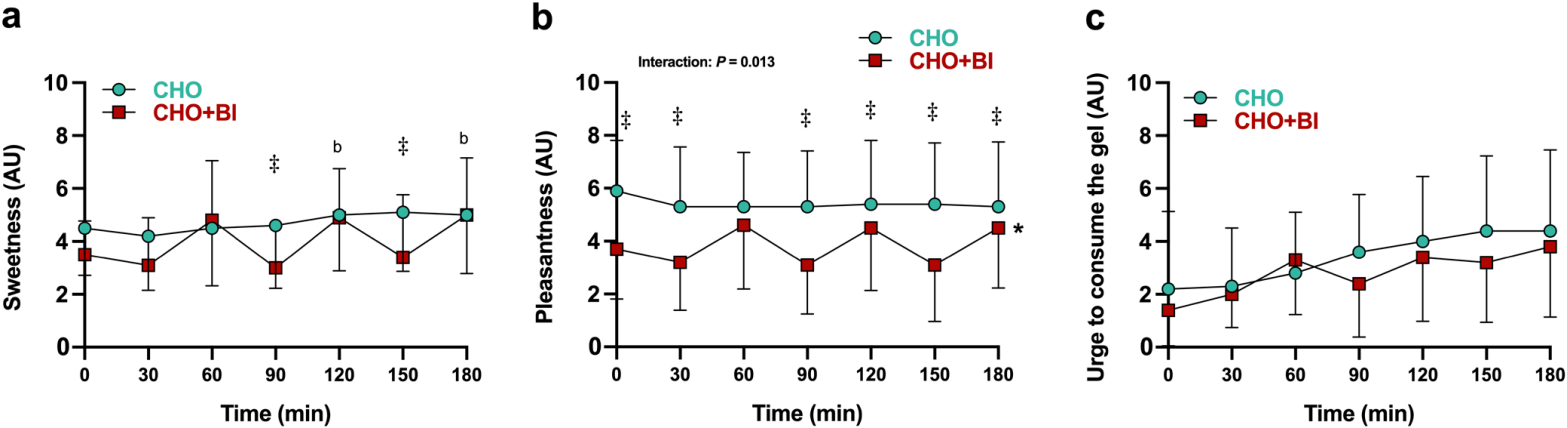
Gel sweetness (a), gel pleasantness (b), and urge to consume the gel (c) in the CHO and CHO + BI trials during the prolonged steady‐state cycle. Data are means ± SD for all figures. *Significantly different from CHO (condition effect), *p* < 0.05. ^‡^Significantly different between conditions at the specified timepoint, *p* < 0.05.

Perception of gel sweetness was not affected by BI ingestion (condition effect, *f* = 3.22, *p* = 0.106, *pη* ^*2*^ = 0.26), but there was a significant timepoint (*f* = 6.56, *p* = 0.002, *pη* ^*2*^ = 0.42) and interaction effect (*f* = 4.84, *p* = 0.013, *pη* ^*2*^ = 0.35), which coincided with the timing of ingestion of the BI gels, with CHO + BI perceived as less sweet than CHO at 90 and 150 min (both *p* < 0.05; Figure 7a). Gel pleasantness was significantly lower in CHO + BI compared with CHO (*f* = 13.61, *p* = 0.005, *pη* ^*2*^ = 0.60), with pairwise differences observed at all timepoints except at 60 min (all *p* < 0.05; Figure 7b). No significant effect of timepoint was observed (*f* = 2.13, *p* = 0.148, *pη* ^*2*^ = 0.19). Finally, the urge to consume a gel increased significantly across timepoints (*f* = 6.27, *p* = 0.014, *pη* ^*2*^ = 0.41), with no differences between conditions (Figure 7c). There were no differences in total fluid intake between conditions (CHO: 2007 ± 490 mL, CHO + BI: 2095 ± 456 mL; *p* = 0.563).

## 4 Discussion

The primary aim of our study was to examine the effects of BI and CHO co‐ingestion during exercise on sprint and TT performance in a fatigued state in trained cyclists. This is the first study to show that ingestion of 20 g of BI in a novel gel format during exercise improves 12‐min TT cycling performance ~6% following 3 h of moderate‐intensity cycling compared to CHO feeding alone. Of note, even BI and CHO co‐ingestion was not sufficient to prevent the fatigue‐induced decline in TT performance compared to a non‐fatigued state. In contrast, sprint and 2‐min TT performance were unaffected by BI ingestion. Importantly, the ergogenic effects of BI co‐ingestion occurred with minimal GI discomfort and without altering whole‐body substrate utilization, energy expenditure, or oxygen cost during exercise. Collectively, these data suggest that BI and CHO co‐ingestion during exercise may represent a viable strategy for practitioners and athletes seeking to enhance high‐intensity exercise performance in a state of accumulated fatigue.

CHO ingestion at a rate of 80 g·h^−1^ did not negate the reduction in 12‐min TT (~12% decline) observed after 3 h of moderate‐intensity cycling. This is not necessarily surprising given that some level of physiological deterioration is to be expected. Previous research [8, 10] has shown that CHO ingestion limits, but does not abolish, the decline in parameters of the power‐duration relationship after prolonged, fatiguing exercise. Furthermore, BI and CHO co‐ingestion conferred a ~6% improvement in 12‐min TT performance compared with CHO alone, supporting the notion that the state of alkalosis achieved by the end of the 3‐h exercise attenuated the metabolic perturbations that otherwise compromise high‐intensity exercise performance in a fatigued state. The magnitude of this improvement may seem surprising given that previous studies have reported smaller performance benefits, in the region of 1%–3% [14, 20, 32], following the ingestion of BI. These comparisons should be interpreted in light of the study design, as we examined the effects of BI and CHO co‐ingestion on performance in a fatigued state, rather than during fresh, isolated high‐intensity efforts. The greater improvement observed in the present study may (at least partly) reflect the amplified role of extracellular buffering when physiological characteristics begin to deteriorate. We observed a performance benefit of BI co‐ingestion in the 12‐min TT but not in the sprints or 2‐min TT, which could be due to test sequencing whereby BI ingestion may improve high‐intensity repeatability or simply pacing effects inherent to a fixed‐order protocol. Importantly, BI may influence aerobic exercise performance through mechanisms other than extracellular buffering. Intravenous BI infusion has been shown to attenuate exercise‐induced arterial oxygen desaturation during maximal exercise [33], and this could also contribute to the ergogenic effects observed following prolonged endurance exercise.

The absolute increase in HCO_3_^−^ from baseline to individual peak (~6 mmol·L^−1^) is consistent with the 4–8 mmol·L^−1^ increases following pre‐exercise SB ingestion at doses of 0.2–0.3 g·kg^−1^ [13, 34] and falls above the threshold of > 5 mmol·L^−1^ suggested to lead to ergogenic effects [16, 35]. The delta in peak HCO_3_^−^ concentration observed in our study was, however, on the lower end of the range reported in the literature, and this may be explained by several factors. Firstly, even though BI ingestion was front‐loaded to the initial portion of the 3 h cycle, the dose was split over four timepoints (0, 30, 90, and 150 min) as opposed to the administration of a single bolus; secondly, the ingestion of CHO alongside BI possibly delayed BI absorption, given that increasing the energy density of an ingested solution decreases the rate of gastric emptying [36]; thirdly, the administration of BI during exercise adds an additional layer of complexity, due to the unknown interaction between exercise‐induced physiological perturbations and BI absorption kinetics, which may have attenuated the magnitude of alkalosis compared with pre‐exercise protocols designed around individualized time to peak HCO_3_^−^ concentrations; and finally, the absolute dose of 20 g resulted in interindividual variation in relative doses (0.23–0.35 g·kg^−1^), with the participants receiving the lowest doses (0.23–0.25 g·kg^−1^) exhibiting the smallest increases in HCO_3_^−^ (2.80–5.20 mmol·L^−1^). Interestingly, despite the lower dose, three of the five participants demonstrated improvements in 12‐min TT performance of at least 5%. This somewhat challenges the idea of the existence of a 5 mmol·L^−1^ ergogenic threshold.

Interindividual variability in the response to SB supplementation is well documented [37], and the present study was no exception. In this study, there were three participants who did not see an improvement in 12‐min TT performance with BI ingestion. Of these, two participants showed no decline in power output in the 12‐min TT between non‐fatigued and fatigued states. Although this may indicate limited fatigue development in these individuals at this level of accumulated work, the absence of protocol‐specific reliability data prevents us from excluding normal measurement variability as a contributing factor. The third participant demonstrated an identical decline in 12‐min TT between both conditions and also exhibited the smallest ΔHCO_3_^−^ of all the participants (2.8 mmol·L^−1^) in the BI condition, which may reflect the comparatively lower relative dose received (0.24 g·kg^−1^), differences in absorption kinetics (whereby true peak HCO_3_^−^ concentrations had not yet been reached at the onset of the performance tests), or a true absence of an ergogenic effect in this individual.

The recovery of acid–base balance between repeated high‐intensity efforts may be an important contributor to the preservation of the parameters of the power‐duration curve in a fatigued state. In this sense, whether BI ingestion is able to facilitate this recovery merits investigation [38, 39]. Our power profiling battery was designed to capture efforts spanning different durations and energy system contributions along the power‐duration curve. As already mentioned, no between‐condition differences were observed for the sprints or 2‐min TT, with the ergogenic effect of BI only emerging during the 12‐min TT. Previous research [40] has shown that performing 3 × 10‐s maximal sprints dampens subsequent 4‐km time‐trial performance, due to earlier utilization of buffering capacity during the preceding sprints. Given the relatively short recovery period (5 min) between efforts, it is possible that a similar mechanism hindered performance in the 2‐min TT. However, this cannot be verified, as HCO_3_^−^ was not sampled prior to commencing this effort, and HCO_3_^−^ at the subsequent timepoint (PT2; post‐2‐min TT) remained higher in CHO + BI than CHO only, which does not clearly support a depleted buffering response at the onset of the 2‐min TT. Another possible explanation could be the lower sensitivity of shorter efforts to acid–base status. Equally, the improvement observed in the 12‐min TT may reflect enhanced high‐intensity exercise repeatability with BI co‐ingestion, which in itself represents an important component of durability in cycling races characterized by repeated maximal efforts in a fatigued state [8, 41, 42], although the present study cannot differentiate whether this improvement reflects improved durability associated with the fatigue accumulated in the 3‐h ride per se, or recovery effects specifically related to the preceding sprints and 2‐min TT efforts within the power profiling test battery. Further research directly assessing the effects of BI supplementation on repeated high‐intensity efforts in a fatigued state, with an appropriate time course analysis, is required to determine if BI co‐ingestion influences recovery under these conditions.

Blood lactate responses in the fatigued power profiling (PT2–PT5) were significantly higher following the ingestion of BI, which is similar to previous studies [14, 40]. The absolute increase from post‐sprints to post‐2 min TT (PT1 to PT2) was significant in CHO + BI (4.1 mmol·L^−1^, *p* = 0.004) but not in CHO (3.1 mmol·L^−1^, *p* = 0.108), and the same was observed between the start and end (PT3 to PT4) of the 12‐min TT (CHO + BI: 4.4 mmol·L^−1^, *p* = 0.032; CHO: 2.9 mmol·L^−1^, *p* = 0.063), albeit somewhat more noticeable at this timepoint. One possible explanation for the elevated lactate response is a higher lactate efflux from contracting muscle, potentially enabled by a greater intra‐extracellular H^+^ gradient following BI ingestion [17, 43]. This would be consistent with reports that exercise‐induced metabolic alkalosis may increase the rate of glycolysis [44], which could plausibly contribute to greater lactate production. Ultimately, the elevated lactate response observed with BI supplementation and the subsequent performance improvement in the 12‐min TT challenge traditional views that lactate accumulation itself directly impairs exercise performance.

SB supplementation is well‐known as a possible cause for GI distress, which can negate its ergogenic effects if symptoms occur during exercise [18]. In our study, GI symptoms were negligible across both conditions, with identical cumulative and individual symptom scores between conditions. While the present study did not directly compare delivery formats, the frequency of GI symptoms using conventional SB formats such as capsules (enteric‐coated or gelatin) [12, 20] or beverages [40] has generally been higher, with participants occasionally reporting severe bloating, belching, diarrhea, and even vomiting. In contrast, other studies have reported minimal GI discomfort in endurance‐trained cyclists when SB was delivered using mini‐tablets, albeit at rest [21] or in isolated repeated high‐intensity time‐trials [22]. More research examining the prevalence of GI symptoms during prolonged endurance exercise is needed. We speculate that the high fluid intake consumed during the BI trial and co‐ingestion with CHO may have reduced the prevalence of GI discomfort. Participants ingested approximately 2 L of water across the 3 h exercise via the standardized gel co‐ingestion (1200 mL of water across all timepoints) and *ad libitum* intake (~850 mL of water), which may have reduced the osmotic gradient created by the hypertonic nature of BI gels and, in turn, attenuated the fluid shift from the plasma into the GI tract [45]. Notably, BI ingestion via the novel gel format did not increase thirst, as there were no differences in *ad libitum* water intake between conditions, in contrast to Gough and Sparks [22], who reported elevated thirst in five participants following ingestion of a hydrogel‐based SB delivery system. Of note, as evidenced by the lower gel pleasantness scores at the timepoints corresponding to the ingestion of BI, some participants preferred to have the gel diluted in water prior to ingestion due to its dense texture. This may represent a simple yet practical strategy to improve palatability and delivery during exercise.

This study is not without its limitations. Due to the distinct taste and texture profile of the BI gel, a double‐blinded study design was not possible, and we therefore cannot rule out an effect of expectancy on the reported outcomes. Relatedly, the BI dose was not individually adjusted to body mass due to the delivery format used (i.e., gels), resulting in relative doses ranging from 0.23 to 0.35 g·kg^−1^ across participants. This is reflected in a strong positive correlation between relative BI and the magnitude of change in HCO_3_^−^ concentrations (*r* = 0.778), and hence the varied blood acid–base responses observed. Despite this, neither relative dose nor ΔHCO_3_^−^ were significantly associated with the magnitude of performance improvement in the 12‐min TT, which is most likely attributable to the small sample size limiting the statistical power to detect such a relationship. Furthermore, the findings of this study quantify the additional effect of the BI formulation when co‐ingested with CHO and should not be interpreted as the effect of BI in the absence of CHO feeding, which would likely have resulted in impaired performance and potentially an inability to complete the 3‐h steady‐state portion of the trial, given the well‐documented consequences of hypoglycaemia during prolonged endurance exercise [46, 47]. The use of indirect calorimetry to estimate CHO and fat oxidation rates may also present a methodological challenge in SB research, as standard stoichiometric equations rely on the assumption that expired V̇CO_2_ reflects metabolic V̇CO_2_ production only. SB‐induced buffering of H^+^ may, however, also augment non‐metabolic V̇CO_2_ production and thus increase V̇CO_2_ irrespective of substrate oxidation. If we assume complete buffering of the ingested dose (20 g) and take into account the V̇CO_2_ observed in the experimental trials (~2.5 L·min^−1^ on average), the non‐metabolic contributions would have accounted for a theoretical maximum of ~1% of total V̇CO_2_ production (or 0.03 L·min^−1^), corresponding to a possible overestimation of 0.1 g·min^−1^ in whole‐body CHO oxidation and an underestimation of 0.05 g·min^−1^ in whole‐body fat oxidation rates. As such, we do not consider this to have meaningfully influenced our findings nor its interpretation. Additionally, the small sample size and heterogeneity of the participant cohort may limit the generalisability of the findings at this stage. Finally, this study does not provide evidence for the superior ergogenic effects of adopting BI supplementation strategies during exercise compared with more traditional approaches. A direct comparison between pre‐exercise loading and during‐exercise ingestion protocols, using this delivery format or others, and matched relative BI doses, is needed to determine whether both strategies produce identical alkalotic and ergogenic responses, and under what training and racing scenarios one format might be preferred over another. With professional cyclists and runners reportedly adopting fuelling strategies exceeding 100 g·h^−1^ of CHO, future investigations may wish to examine the dose–response of BI and CHO co‐ingestion during exercise and its effects on individual time to peak HCO_3_^−^ concentrations.

### 4.1 Perspectives

We demonstrate that an absolute BI dose spread out over 3 h of cycling is effective at inducing metabolic alkalosis and improving subsequent performance. From a practical standpoint, the novel gel format may offer an advantage over traditional SB delivery methods, which are less suited to ingestion during exercise due to palatability and/or logistical constraints of swallowing multiple capsules mid‐effort. Energy gels are already widely used by endurance athletes during competition, meaning that this strategy could, in principle, be integrated with established race‐day fueling practices. This preliminary work opens up a potential avenue for its application across a range of endurance sports where prolonged exercise precedes decisive high‐intensity efforts, pending confirmation in larger cohorts, elite athlete populations, and real‐world competitive settings. Endurance athletes and applied practitioners may wish to consider trialing BI gels as part of existing fueling strategies, either as a standalone during‐exercise ingestion strategy or combined with a pre‐exercise loading phase. In practice, should the suitability of this delivery format be confirmed using real‐world fatiguing exercise protocols incorporating mixed‐intensity efforts representative of competitive demands, the distribution of BI gels could be evenly spread throughout exercise as a top‐up strategy to maintain pre‐exercise HCO_3_^−^ concentrations (e.g., one BI gel every hour), or strategically timed at a specific portion of the race to allow sufficient time for peak alkalosis to occur before a decisive effort (e.g., three to four BI gels ingested across a 60–80 min window prior to a critical climb), thereby maximizing buffering capacity at the most pivotal moment of the race. These proposed strategies remain hypothetical at this stage and represent clear directions for future research.

## 5 Conclusion

The present study demonstrated, for the first time, that BI and CHO co‐ingestion during prolonged exercise via a novel energy gel format is an effective strategy to achieve blood alkalosis and improve 12‐min TT performance, with only very mild GI discomfort. These findings suggest that the ergogenic potential of BI may extend beyond isolated high‐intensity exercise performed in a fresh state to high‐intensity cycling performance following prolonged exercise‐induced fatigue. From an applied perspective, the novel gel format is a practical and ecologically valid approach to delivering BI during prolonged exercise. Whether it should be implemented as a standalone strategy or as a top‐up to a pre‐exercise loading phase remains to be investigated.

## Funding

This work was supported by the Portuguese Foundation for Science and Technology (FCT) through the individual doctoral grant 2023.00585.BD for the first author.

## Conflicts of Interest

Nutrition products for the experimental trials were provided by MNSTRY (Freudental, Germany). The EPOC blood analyzer and associated consumables for blood acid–base balance and electrolyte analysis were provided by Siemens Healthineers (Erlangen, Germany). MNSTRY and Siemens Healthineers had no role in the study design, data collection, analysis, interpretation, or decision to publish.

## Supporting information


**Figure S1:** Sodium (a), potassium (b), calcium (c) and chloride (d) responses to 180‐min of moderate‐intensity cycling and a power profiling protocol in a fatigued state, with blood sampling taken at 0, 60, 120 and 180 min during the steady‐state cycle, and at post 3 × 6‐s sprints (PT1), post‐2 min TT (PT2), prior to the 12‐min TT (PT3), post‐12 min TT (PT4) and after a 10‐min passive recovery period (PT5). Data are means ± SD for *n* = 11 trained cyclists. *Significantly different from CHO (condition effect), *p* < 0.05. ^‡^Significantly different between conditions at the specified timepoint, *p* < 0.05. ^^^Significantly different from previous timepoint (time effect), *p* < 0.05.

## Data Availability

The data that support the findings of this study are available from the corresponding author upon reasonable request.
