# Supplementary material for: Bicarbonate and Carbohydrate Co‐Ingestion During Exercise in a Novel Gel Format Improves Time‐Trial Performance After 3 h of Moderate‐Intensity Exercise in Trained Cyclists
Source: Scand J Med Sci Sports. 2026 Sep 25;36(10):e70373. doi: 10.1111/sms.70373 (PMC13614109; doi:10.1111/sms.70373)
Supplement: Supplementary file 1 [file SMS-36-e70373-s001.docx]

**(Suppl) Figure 1**. Sodium (a), potassium (b), calcium (c) and chloride (d) responses to 180-min of moderate-intensity cycling and a power profiling protocol in a fatigued state, with blood sampling taken at 0, 60, 120 and 180 min during the steady-state cycle, and at post 3 x 6-s sprints (PT1), post-2 min TT (PT2), prior to the 12-min TT (PT3), post-12 min TT (PT4) and after a 10-min passive recovery period (PT5). Data are means ± SD for *n* = 11 trained cyclists. *Significantly different from CHO (condition effect), *P* < 0.05. ^‡^Significantly different between conditions at the specified timepoint, *P* < 0.05. ^^^Significantly different from previous timepoint (time effect), *P* < 0.05.

Blood Na^+^ concentrations (Suppl Figure 1a) were significantly higher in CHO+BI compared with CHO (condition effect, *f* = 46.97, *P* < 0.001; MD: 2.67 mmol·L^–1^, 95% CI: 1.90, 3.43) and changed significantly over time (*f* = 4.03, *P* < 0.001), with concentrations peaking at PT1 before declining towards PT5. No significant condition × timepoint interaction was observed (*f* = 1.35, *P* = 0.221). Blood K^+^ concentrations (Suppl Figure 1b) also changed significantly over time (*f* = 5.66, *P* < 0.001) but were unaffected by the experimental conditions (*f* = 1.89, *P* = 0.171), with no significant condition × timepoint interaction (*f* = 1.25, *P* = 0.276).

Blood Ca^2+^ concentrations (Suppl Figure 1c) were significantly lower in CHO+BI compared with CHO across all timepoints (condition effect, *f* = 197.37, *P* < 0.001; MD: 0.08 mmol·L^-1^, 95% CI: 0.07, 0.09), with significant between-condition differences observed at every timepoint from 0 min through to PT5 (all *P* ≤ 0.026). A significant time effect (*f* = 10.00, *P* < 0.001) and condition × time interaction (*f* = 2.37, *P* = 0.020) were also observed.

Blood Cl⁻ concentrations (Suppl Figure 1d) were significantly higher in CHO compared with CHO+BI (condition effect, *f* = 12.72, *P* < 0.001; MD: 1.82 mmol·L^-1^, 95% CI: 0.81, 2.83) and changed significantly over time (*f* = 4.51, *P* < 0.001). A significant condition × timepoint interaction was also observed (*f* = 2.78, *P* = 0.007), with between-condition differences during the fatigued power profiling protocol at PT3 (*P* = 0.001, *g* = 1.38), PT4 (*P* = 0.002, *g* = 0.93), and PT5 (*P* = 0.007, *g* = 0.97), but not during the 3 h cycle (all *P* > 0.05).
